# Coronavirus Pandemic: What Nuclear Medicine Departments Should Know

**DOI:** 10.2967/jnmt.120.247296

**Published:** 2020-06

**Authors:** Gopinath Gnanasegaran, Hian Liang Huang, Jessica Williams, Jamshed Bomanji

**Affiliations:** 1 Department of Nuclear Medicine, Royal Free London NHS Foundation Trust, London, United Kingdom; 2 Institute of Nuclear Medicine, University College London Hospital, London, United Kingdom; and; 3 Harley Street Clinic, HCA Healthcare United Kingdom, London, United Kingdom

The new coronavirus pneumonia (coronavirus disease 2019 [COVID-19]) was first reported in Wuhan, China, in December 2019; the virus was extracted from lower-respiratory-tract samples from humans (1). The World Health Organization officially announced COVID-19 to be a pandemic on March 11, 2020 (2).

This novel coronavirus is spreading rapidly despite precautions. The most common symptoms of COVID-19 include severe pneumonia (fever, dry cough, dyspnea) and fatigue (1). Sore throat, headache, loss of taste and smell, rhinorrhea, and diarrhea can occur uncommonly (1). Lymphopenia is common (3,4). The mean incubation period is reported to be 5.2 d, and transmission can occur during that period in asymptomatic patients (5). The virus is reported to be transmitted via respiratory droplets and fomites during unprotected close contact with an infected individual (6,7).

Spread of the infection has been reported in 212 countries and regions (2). Throughout time, humans have encountered epidemics and pandemics, and several of these have changed the course of history. Pandemics increase morbidity and mortality and cause significant economic, political, and social disruption (8).

The aim globally is to encourage physical distancing in order to slow disease transmission and avoid increased strain on local health-care systems. The significant health-care challenges are in the production, supply, and availability of equipment for patient care and staff during this pandemic. A coordinated response and responsibility should be emphasized and implemented on time to maintain public health awareness and information, reduce transmission, and care for and treat the patients with COVID-19 (2). Significant gaps, misunderstandings, and challenges exist in global pandemic preparedness. To compound the problem further, the guidance and recommendations are rapidly changing as new evidence emerges and evolves. Local policy should be adopted in consensus with national and international recommendations. People must follow the recommendations and restrictions of the local government or health department.

Hospitals and departments should have a standard operating procedure in place for staff who image patients suspected or confirmed to have COVID-19, and systems should be in place to ensure that these procedures are regularly updated. This article is based on the currently available literature. Our purpose is to discuss and review precautions and safety measures for nuclear medicine department staff in managing patients with known or suspected COVID-19. The situation is changing rapidly, and there is every chance that discussion stemming from this article will change over the coming days and weeks. The responsibility lies with each institution or hospital to ensure its written policy adheres to that outlined by national public health guidance in its respective country.

## SEVERE ACUTE RESPIRATORY SYNDROME CORONAVIRUS 2

COVID-19 is caused by a novel β-coronavirus (1) that has been given the name “severe acute respiratory syndrome coronavirus 2” (SARS-CoV-2) (2). It belongs to the Coronaviridae family and is an enveloped positive-strand RNA virus (1,9). Coronaviruses are named for the crownlike spikes on their surface (9,10). The most likely origin of the novel coronavirus is zoonotic, given it has a genome 96% identical to that of a severe acute respiratory syndrome–like coronavirus found in bats (6,11). The novel virus has been detected in respiratory, fecal, and blood specimens of infected patients (6,11) and is reported to remain viable as an aerosol for up to 3 h (12). There are reports that transmission can occur via ocular surfaces, as infected droplets and bodily fluids might contaminate the human conjunctival epithelium (13). The virus was reported to be found in upper respiratory samples 1–2 days before the onset of symptoms (14) and is thought to be spread mainly via asymptomatic carriers (5,15,16).

## NUCLEAR MEDICINE STAFF AND PATIENTS

In terms of personal protective equipment (PPE), the World Health Organization recommends taking contact and droplet precautions before entering the room of a suspected or confirmed COVID-19 patient. These include wearing disposable gloves to protect the hands; a clean, nonsterile, long-sleeved gown to protect clothing; medical masks to protect the nose and mouth; and eye protection such as goggles or a face shield (17,18). Respirators (e.g., N95) are recommended for aerosol-generating procedures (17). With the increasing number of cases and the shortage of testing kits for COVID-19, there should be greater emphasis on infection-control and social-distancing measures for both the public and staff members in the health-care environment. Effective and efficient use of both staff and equipment in nuclear medicine departments is crucial for patient care and workplace safety. Several national and international bodies have reported numerous measures that might be implemented nationally and regionally (2,17–19). However, the policies and their implementation will vary from region to region. Departments should be aware of their national or local hospital policies and follow them accordingly. Numerous articles about radiology procedures on COVID-19 patients have appeared, but there is limited advice and information related to nuclear medicine services. Compared with conventional radiological imaging, the requirements and logistics for nuclear medicine imaging are relatively complex, such as scheduling appointments, contacting patients, maintaining regulatory compliance, prioritizing procedures, limiting the duration of scans, and preventing infection (Table 1) (20–25). Our top priorities should be ensuring the personal wellness of our staff and providing sufficient training and staff coverage to manage patients with suspected or confirmed COVID-19.

**TABLE 1:** Consensus Guidance for Nuclear Medicine Departments, Staff, and Patients (6,17,20–25)

Managing nuclear medicine department	Nuclear medicine staff	Patients attending nuclear medicine center
Promote and practice social distancing (2 m or 6 ft)	Train in infection control	Screen patients and visitors before they enter department
Assess risk at local level, with local context taken into consideration (should not replace or reduce ability to provide optimal patient or staff safety)	Promote and practice social distancing (2 m or 6 ft)	Make initial risk assessment of patient by phone, when possible
Coordinate transmission of information between hospital information control department and nuclear medicine department	Minimize crowding in workplace (e.g., tea or lunch breaks)	Ask patients to inform nuclear medicine department if patient or family members develop symptoms before scheduled appointment
Ask referring clinicians to clearly indicate whether scans are urgent or nonurgent when requesting them	Maintain (6 ft or 2 m) distance in all patient and staff interactions when possible	Display posters in department reception area to promote hand washing and good respiratory hygiene measures
Train all staff members to ensure maximum compliance and vigilance in line with local guidance	Consider need for contact and droplet precautions (based on nature of task being undertaken)	Promote social distancing (2 m or 6 ft)
Provide clear guidance to staff on how to proceed when patient COVID-19 status is unknown and COVID-19 is circulating at high levels	Practice strict hand hygiene, which should be extended to exposed forearms, after removing any element of PPE	Recommend patient use of fluid-resistant surgical face mask (to minimize dispersal of respiratory secretions and to reduce both direct transmission risk and environmental contamination)
Establish local policy to reschedule nonurgent appointments	Have access to PPE	Ask patients to maintain strict hand hygiene
Display posters to promote hand washing and good respiratory hygiene measures within department	Train on donning and doffing PPE	Ask patients to minimize accompanying visitors and patient escorts
Allocate or make provision for separate space for patients with suspected or known COVID-19 status	Put on appropriate PPE before providing care	Give patients telehealth option (teleclinics to provide reassurance and guidance)
Develop clear escalation pathway to ensure cases are identified in timely manner and triaged	Know what PPE staff should wear for each setting and context	Inform and reschedule nonurgent appointments
Implement stringent local hospital policy for screening of staff, patients, and visitors before they enter department	Adopt single-use policy for gloves and aprons	Inform and reschedule elective therapies
Implement stringent local hospital policy to minimize nonessential visitors in department	Take regular breaks and rest periods	Ensure that patients spend minimum time in department (do not allow patients to remain for long periods in waiting area)
Provide PPE for staff and patients (because of concern about asymptomatic transmission of COVID-19)	Remain connected with rest of staff or with friends and family via group email, e-portal or social media	
Make sure supplies are available, and check stock every day and during day (centralize storage and distribution)	Make sure nuclear medicine physicians or radiologists are familiar with CT appearance of COVID-19	
Implement robust policy for cleaning and decontaminating imaging equipment	Check PET/CT and SPECT/CT scans for CT changes in lungs before sending patient home	
Explore options and encourage reporting of scans from remote sites or home, whenever feasible, according to local policy	Be supportive and caring; nominate staff to look after staff well-being	
Encourage use of virtual conference tools for multidisciplinary or educational meetings		
Provide for flexible staff-schedule rotation (on-site and off-site work, work in small groups)		
Provide relevant, regular, and reliable updates daily		
Develop contingency and business continuity plan		

These are examples based on consensus only, and responsibility lies with each institution or hospital to ensure its written policy adheres to that outlined by national public health guidance in its respective country and hospital.

The team should be made aware that there are asymptomatic carriers of the virus, and a good contact history is of use. We should also ensure that, in the waiting areas, patients have access to alcohol gel, hand-washing facilities, tissue boxes, and masks. Nuclear medicine reception staff should self-protect and be vigilant at all times and encourage patients to self-declare if they or any family members have symptoms or have recently traveled from places affected by COVID-19. The staff should ask specific and direct questions such as about a history of fever, dry cough, dyspnea, and fatigue. Patients should be encouraged to follow basic hygiene practices (26). The patient waiting area should be large enough for patients to maintain distance while seated, or patient appointments should be scheduled so as to avoid having too many patients in the waiting area at a given time.

In general, the nuclear medicine staff, which includes technologists, nurses, and health-care assistants, are at risk of exposure to COVID-19. Unlike radiologic procedures, nuclear medicine procedures require radiotracer injection, and contact between the staff and patient is essential. In most cases, nuclear medicine procedures are outpatient-based; under limited circumstances, they are inpatient-based. Inpatients will be a combination of oncology and nononcology patients. There is a probability that patients with COVID-19 may be asymptomatic at the time they are in the department for their scan. Furthermore, it is possible that not all inpatients have been tested for COVID-19 before they are sent to the department for their scan. These scenarios pose a risk for all staff, from the reception area to the scanning room. In general, most nuclear medicine scanners are not portable, unlike radiography or ultrasound devices; therefore, the need for patients to come to the department for their scan is inevitable. Consequently, we should have a stringent mechanism in place to protect our staff and patients, as well as a contingency plan if staff are temporarily absent because of illness or quarantine, which might affect regular work in the department.

Under the current circumstances, most departments based in hospitals that are COVID-19 hubs are postponing routine elective scans while continuing to provide urgent nuclear medicine scans (e.g., PET/CT scans for oncology patients) (Tables 2–4). Given the widespread transmission and the increased risk of asymptomatic patients, staff should use PPE according to the local policy. The PPE items must be donned before entering the patient area, and the donning and doffing procedure should be performed correctly. In general, the team should minimize the number of staff in each clinical encounter to reduce unnecessary movement into and out of injection or scanning rooms, and staff should wear PPE while escorting patients.

**TABLE 2:** Scheduling Nuclear Medicine Procedures That Use SPECT Tracers

Type of scan (referrals must be reviewed by nuclear medicine consultant)	Scans that can be booked and performed as requested (unless patient is at risk of infection)	Scans that require liaison with clinical team for canceling or rescheduling (inform patient)	Scans that must be postponed or rescheduled (inform patient and clinical team)
Skeletal	Bone scans in cancer patients	Scans for severe pain pre- and postprocedural orthopedic indications (if there is a question of infection, offer ^18^F-FDG PET/CT as alternate)	Scans for pre- and postprocedural orthopedic indications; metabolic bone disease; inflammatory arthropathy
Endocrine	^99m^Tc04 thyroid scans in patients not on antithyroid medications or if question of ectopic or neonatal hypothyroidism		^99m^Tc04 thyroid scans in patients on antithyroid medications; ^99m^Tc-MIBI parathyroid scans for preoperative localization
Cardiovascular (avoid exercise nuclear stress testing because of risk of droplet exposure; consider using pharmacologic stress agents; consider 1-d protocols (e.g., stress–rest)	Myocardial perfusion scans in cases of recent acute coronary syndrome (moderate- to high-risk patients) for urgent coronary revascularization; scans in patients with new or increasing chest pain; scans for preoperative assessment (moderate- to high-risk patients); MUGA scans in oncology patients (before initiation of or subsequent chemotherapy)	Myocardial perfusion scans in patients awaiting liver transplant surgery; scans in patients with stable angina requiring follow-up evaluation; cardiac amyloid DPD scans	^123^I-MIBG heart scans; myocardial perfusion scans in patients awaiting renal transplant surgery; cardiac amyloid DPD scans for follow-up evaluation
Brain			DaTscan (^123^I-FP-CIT) scans
Respiratory (discuss decision to proceed with ventilation–perfusion scan with referrer before booking)	Lung perfusion scans in pregnant patients; lung shunt scans for ^90^Y-SIRT	Ventilation–perfusion scans in patients with pulmonary hypertension or chronic PE on treatment	Ventilation–perfusion scans if question of resolution of PE in patients receiving thromboprophylaxis
Gastrointestinal	Gastrointestinal-bleed Meckel scans		Gastric-emptying esophageal transit scintigraphy; gastroesophageal reflux scintigraphy; SeHCAT small-bowel or colonic transit scans
Hepatobiliary	HIDA scans in patients with biliary leak	HIDA scans if question of acute cholecystitis	Liver or spleen scans; HIDA scans in patients with, for example, cystic duct syndrome or sphincter-of-Oddi dysfunction; liver SPECT in patients with hemangioma; ^99m^Tc-denatured RBC scans
Genitourinary	^99m^Tc-DMSA scans in patients with radiotherapy to abdomen or prior renal surgery; ^99m^Tc-MAG3 scans in patients with urinary leak or transplant rejection; testicular scans in patients with torsion	MAG3 scans if question of obstruction; DMSA scans for donor assessment	^99m^Tc-MAG3 scans for routine follow-up; ^99m^Tc-DMSA scan for follow-up; captopril renogram scans
Infection or inflammation		Scans if question of sepsis in COVID-19–negative patients (suggest FDG PET/CT); scans if question of infection of prosthesis	
Lymphatic system	Sentinel lymph node injections and scans		Lymphoscintigram scans if question of lymphedema
Oncology	^111^In-pentetreotide and ^99m^TcEDDA/HYNIC-Tyr3-Octreotide scans before PRRT	Octreotide/Tektrotyd scans in patients with NET; ^123^I-MIBG scans in patients with pheochromocytoma or paraganglioma	
Miscellaneous	GFR studies in oncology patients before initiation of or subsequent chemotherapy		Dacryoscintigraphy scans; salivary gland scintigraphy; DXA scans

Referrals must be reviewed by nuclear medicine consultants or in multidisciplinary setting. These are examples based on consensus only, and responsibility lies with each institution or hospital to ensure its written policy adheres to that outlined by national public health guidance in its respective country and hospital.

^99m^Tc04 = ^99m^Tc-pertechnetate; MIBI = methoxyisobutylisonitrile; MIBG = metaiodobenzylguanidine; DPD = 3,3-diphosphono-1,2-propanodicarboxylic acid; [^123^I]β-CIT = [^123^I]2β-carboxymethoxy-3β-(4-iodo-phenyl)tropane; [^123^I]FP-CIT = [^123^I]N-ω-fluoropropyl-2β-carbome-thoxy-3β-(4-iodophenyl)nortropane; MUGA = multigated acquisition; SIRT = selective internal radiation therapy; PE = pulmonary embolism; HIDA = hepatobiliary iminodiacetic acid; SeHCAT = selenium homocholic acid taurine; GFR = glomerular filtration rate; MAG3 = mercaptoacetyltriglycine; DMSA = dimercaptosuccinic acid; PRRT = peptide receptor radionuclide therapy; NET= neuroendocrine tumor; DXA = dual-energy X-ray absorptiometry; RBC = red blood cells.

**TABLE 3:** Scheduling Nuclear Medicine Studies That Use PET/CT

Type of PET/CT scan (referrals must be reviewed by nuclear medicine consultant)	Scans that can be booked and performed as requested (unless patient is at risk of COVID-19 infection)	Scans that require liaison with clinical team for canceling or rescheduling (inform patient)
Oncology	^18^F-FDG for staging, restaging, response assessment, and radiotherapy planning	^18^F-FDG, ^68^Ga-DOTATATE/DOTATOC, ^18^F-PSMA, ^68^Ga-PSMA, ^18^F-choline, ^18^F-NaF, or ^18^F-DOPA for follow-up evaluation
	^18^F-PSMA, ^68^Ga-PSMA, or ^18^F-choline for biochemical recurrence	
	^68^Ga-DOTATATE/DOTATOC for staging, restaging, and selecting patients for PRRT	
	^18^F-NaF for bone metastases	
	^18^F-DOPA for diagnosis and staging	
Nononcology	^18^F-FDG for pyrexia of unknown origin in COVID-19–negative patients, for sepsis, for viability testing in symptomatic patients awaiting CABG, for suspected device or prosthetic infection, or for cardiac sarcoidosis	^18^F-FDG for known sarcoidosis in patients on treatment, for polymyalgia rheumatica, or for follow-up of known cardiac sarcoidosis in patients on treatment

These are examples based on consensus only, and responsibility lies with each institution or hospital to ensure its written policy adheres to that outlined by national public health guidance in its respective country and hospital.

FDG = fluorodeoxyglucose; PSMA = prostate-specific membrane antigen; PRRT = peptide receptor radionuclide therapy; DOPA = 3,4-dihydroxyphenylalnine; CABG = coronary artery bypass grafting; NaF = sodium fluoride.

**TABLE 4:** Scheduling Radionuclide Therapy (20,22)

Therapies might be performed as scheduled. However, each patient must be assessed individually by clinical team or MDT prior to scheduling	Therapy requiring cancellation or rescheduling –each patient must be assessed individually, followed by liaison with clinical team or MDT. Inform patient
^177^Lu-DOTATATE peptide receptor radionuclide therapy for metastatic neuroendocrine tumors (consider marrow depletion after procedure)	^131^I therapy for thyroid cancer (follow thyroid cancer management guide for various risk categories)
Selective internal ^90^Y radioembolization therapy for hepatocellular carcinoma or liver metastases	^131^I therapy for benign thyroid disease (most treatments can be postponed; give consideration to patients who cannot tolerate antithyroid medication)
^131^I-metaiodobenzylguanidine therapy for metastatic pheochromocytoma or paraganglioma	Radiosynovectomy for arthritis, hemophilia, and similar conditions
^177^Lu-prostate-specific membrane antigen therapy for metastatic prostate cancer	
^225^Ac-prostate-specific membrane antigen therapy for metastatic prostate cancer	
^223^Ra therapy for prostate cancer with skeletal metastases (consider comorbidities)	

Referrals must be reviewed by nuclear medicine consultants or in multidisciplinary setting. These are examples based on consensus only, and responsibility lies with each institution or hospital to ensure its written policy adheres to that outlined by national public health guidance in its respective country and hospital.

Airborne transmission of COVID-19 continues to be debated. There is an ongoing dilemma on whether to do ventilation–perfusion scans. It is reported that airborne viruses can spread in air-conditioning and ventilation systems. Medical procedures associated with the generation of aerosols, such as ventilation scans and oxygen supplementation, might carry an increased risk of transmission. Therefore, some have suggested stopping ventilation–perfusion scan services because the ventilation scan is aerosol-based. In addition, the use of perfusion-only scans is unlikely to be of any benefit if COVID-19 is suspected, as the COVID-19 response might alter the macroaggregated albumin distribution (20).

Others have proposed several alternatives, such as performing only perfusion imaging in, for example, pregnant patients or performing perfusion SPECT or SPECT/CT. Overall, it depends on the local conditions. Decisions should be based on national or regional policies (22), and special precautions, especially for personnel conducting these tests, must be taken. A chest radiograph should be mandatory before a ventilation–perfusion scan. The current reports suggest that asymptomatic COVID-19 carriers may have positive chest radiography results after 14 d of quarantine, even with no reverse-transcription polymerase chain reaction testing for COVID-19 (27). The chest radiography findings in COVID-19 patients are reported to frequently show bilateral lower-zone consolidation (peaking at 10–12 d from symptom onset) (28).

## RADIONUCLIDE THERAPIES

Nuclear medicine departments perform various radionuclide therapies for both benign and malignant disease. The hospital and department providing these services should have a practical and realistic solution. The multidisciplinary team (MDT) must make a pivotal decision to continue or stop therapy service temporarily. The radionuclide therapy service depends on multiple factors, such as whether the treatment is outpatient or inpatient, the availability of beds for inpatient-based treatments, regular supply and delivery of radiopharmaceuticals, the risk of a patient contracting COVID-19 during the hospital stay, the staff skill mix (in case therapy staff is infected with the virus and substitute staff must be enlisted), robust selection criteria, and treatment of elderly cancer patients with comorbidities. Finally, when patients are treated, they should additionally consent to the risk of COVID-19 during their stay in the hospital, and the need for radionuclide therapy should balance against the risk of contracting COVID-19 (Table 4).

## SOCIAL DISTANCING

Physical isolation or distancing of staff from one another is crucial to prevent transmission from asymptomatic carriers. In the scan-reporting rooms, it is suggested that the workstations be separated by at least 2 m or 6 ft (23–25). First, the department should consider providing alternative technologic solutions that allow remote or off-site work for nuclear medicine consultants and residents (e.g., reporting of scans and protocolling procedures). Second, multidisciplinary meetings or case discussions should be web-based or teleconferenced (23–25). Several departments have opted for flexible rotations or schedules, such as by working in small teams or by working for 1 wk on-site and then 1 wk remotely. Establishing a group email list or a social media group to keep in touch and communicate effectively is essential.

## IMAGING EQUIPMENT

Local hospitals should have clear policies and procedures in place for nuclear medicine staff who image suspected or confirmed COVID-19 patients. The standard operating procedures of the department or health-care system should be updated regularly as evidence evolves. van Doremalen et al. (12) have studied how long the virus survives in the air and on surfaces. They confirmed that the novel coronavirus remained active for 48–72 h on plastic and stainless steel surfaces, 24 h on cardboard, and 4 h on copper (12). However, these times will vary under real-world conditions and might depend on the temperature, humidity, ventilation, and amount of virus deposited (12).

**FIGURE 1.**
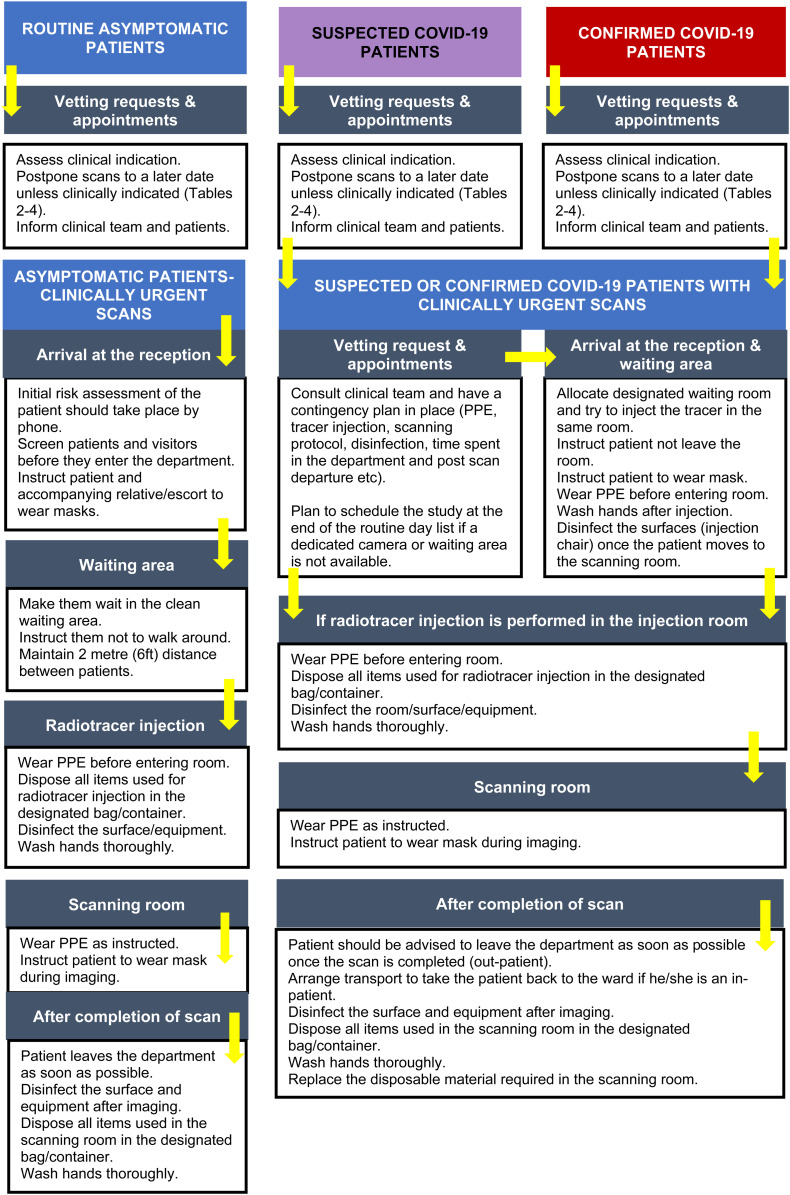
Basic contingency for nuclear medicine imaging. (Adapted from (41).)

The most important COVID-19 factors related to nuclear medicine include clean imaging techniques and decontamination of imaging equipment (e.g., SPECT/CT and PET/CT scanners), in addition to decontamination of any surface that may have come into contact with respiratory droplets. In general, after the patients are scanned, the scanner and room surface should be disinfected to prevent potential spread, and appropriate training of environmental maintenance staff is recommended (Fig. 1) (20). Public Health England has published guidance entitled, “COVID-19: Cleaning in Non-Healthcare Settings” (29). The risk of infection depends on several factors, such as the type of surfaces contaminated, the amount of virus shed from the individual, the time the individual spent in the setting, and the time since the individual was last in the environment (29). All surfaces that the symptomatic person might have come into contact with must be cleaned and disinfected (e.g., visible body fluids, imaging equipment, chair, bathrooms, door handles, telephones, and grab-rails in corridors and stairwells) (29). The PPE should be worn for cleaning an area where a person with possible or confirmed COVID-19 has been (29). Public Health England recommends the use of a combined detergent disinfectant solution at a dilution of 1,000 parts per million available chlorine; if an alternative disinfectant is used within the organization, this alternative should be checked to ensure that it is effective against enveloped viruses (29).

The British Society of Thoracic Imaging has produced action cards to assist with designing local radiology standard operating procedures for patients who have or are at risk of COVID-19 (e.g., transferring a patient to a CT scanner or performing a CT scan) (30). These action cards might be applied to nuclear medicine departments as well. However, they are examples only, and the responsibility lies with each institution or hospital to ensure that its written policy adheres to the national public health guidance in the respective country.

## RADIOPHARMACEUTICALS

The functioning of nuclear medicine procedures depends on the availability of radioisotopes and kits. These are not always locally produced; nuclear medicine centers might have to rely on obtaining them from national or international supply-and-distribution channels. In the current scenario, with land and air traffic lockdowns, a shortage of radioisotopes and kits is expected, and it is difficult to predict when the shortage will occur or for how long. For efficient use of kits, block booking of specific procedures should be envisaged. Alternately, PET/CT scans can be used in place of single-isotope methods for some indications (e.g., bone imaging with ^18^F-NaF and infection imaging with ^18^F-FDG). Myocardial perfusion imaging can be performed as a 1-d protocol (stress–rest). Local radiopharmacists or managers of nuclear medicine departments should contact the suppliers and update the local team so that bookings can be planned accordingly. In comparison to SPECT services, PET centers with local cyclotrons might continue to function as usual in most cases. For departments without cyclotrons, the availability of ^18^F-FDG will depend on local conditions.

## STAFF WELL-BEING

The current scenario might cause psychologic distress, social insecurity, and financial insecurity. Staff coming to work at the hospital are concerned that they might contract the virus and expose their friends or family. We should try to provide relevant and reliable information to allay their fears (e.g., social distancing, infection control, and self-quarantine). There should be specific local guidelines for viral testing of staff returning to work after illness. The team should remain connected with one another or with their friends and families by such means as group email, e-portals, and social media.

## CONTINUING MEDICAL EDUCATION AND PROFESSIONAL DEVELOPMENT

Departments that are active in teaching and training can use online teaching material and webinars, which are available from most national and international nuclear medicine societies, as an alternative to face-to-face interaction and learning. National organizations should make some of their online education material available free to its members. Research work will be challenging during the current circumstances (except for research related to COVID-19), as most institutions have suspended their projects, and alternative ways of collaborating should be envisaged to prevent disruption of vital projects (23,25).

## CHEST FINDINGS FOR PET/CT AND SPECT/CT

**FIGURE 2.**
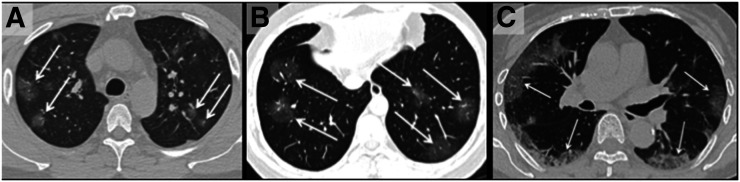
(A) Unenhanced axial CT image from 36‐year‐old man shows bilateral ground‐glass opacities in upper lobes with rounded morphology (arrows). (B) Axial CT image from 44‐year‐old man shows larger ground-glass opacities bilaterally in lower lobes with rounded morphology (arrows). (C) Axial CT image from 65‐year‐old woman shows bilateral ground‐glass and consolidative opacities with striking peripheral distribution. (Reprinted with permission of (32).)

**FIGURE 3.**
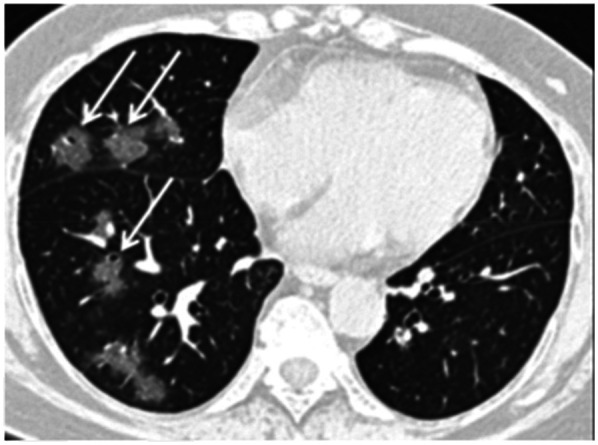
Unenhanced axial CT image from 56‐year‐old woman shows ground‐glass opacities with rounded morphology (arrows) in right middle and lower lobes. Left lung was normal. (Reprinted with permission of (32).)

**FIGURE 4.**
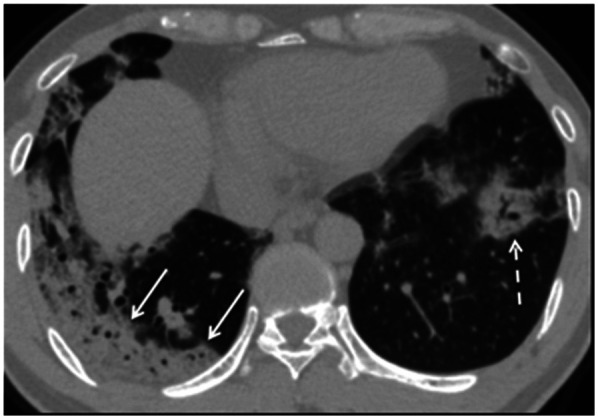
Unenhanced axial CT image from 42‐year‐old man in late time group (10 d from symptom onset to this scan) shows bilateral consolidative opacities with striking peripheral distribution in right lower lobe (solid arrows) and with rounded morphology in left lower lobe (dashed arrow). (Reprinted with permission of (32).)

**FIGURE 5.**
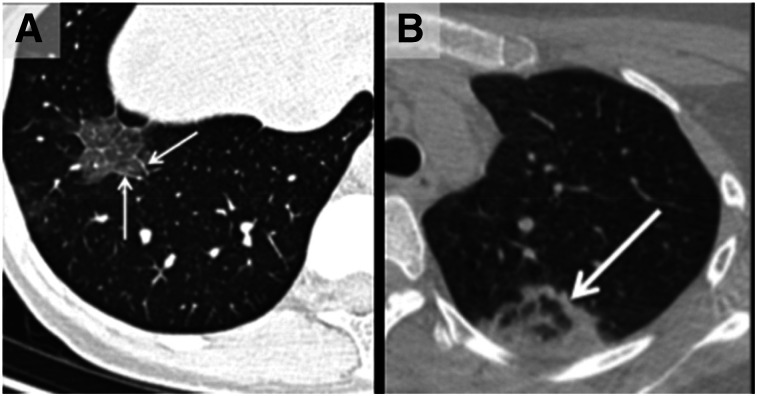
(A) Unenhanced axial CT image from 43-y-old woman shows crazy‐paving pattern as manifested by right-lower-lobe ground‐glass opacification with interlobular septal thickening (arrows) and intralobular lines. (B) Axial CT image from 22‐year-old woman shows area of faint ground‐glass opacification in left upper lobe, with ring of denser consolidation (arrow, reverse halo sign). (Reprinted with permission of (32).)

Incidental parenchymal lung abnormalities on chest images have been reported for patients with COVID-19, and prompt recognition may be useful for timely isolation and treatment (Figs. 2–5) (1). The chest radiographic and CT appearance of COVID-19 has been reported to overlap significantly with the findings for other types of coronavirus infections (31–33).

Chest CT is reported to be an essential component in the diagnostic algorithm for patients with suspected COVID-19 (32,33). The reported sensitivity of chest CT in detecting COVID-19 at the initial presentation is 56%–98% during the early stages of disease development (34,35), and the specificity is low (25%) (36).

Chest CT has limited sensitivity and a low negative predictive value early after symptom onset and is unlikely to be used as a reliable independent tool to rule out COVID-19 (32).

The initial findings in infected patients from Wuhan have shown bilateral lung opacities. The typical features include lobular and subsegmental areas of consolidation (31,32). Other groups have reported high rates of ground-glass opacities and consolidation, sometimes with a rounded morphology and peripheral lung distribution (31,37). The more extensive disease is reported to be seen on CT approximately 10 d after the onset of symptoms (37).

The frequency of CT findings is related to the infection time course (31,32). On the basis of the current evidence, there are ground-glass abnormalities in the early disease phase, followed by crazy paving and increasing consolidation later in the disease course (32,37).

Multifocal involvement is reported to be common, and the CT signs gradually improve approximately 14 d after symptom onset (31,32,36,37).

The hallmark of COVID-19 on CT is ground-glass opacities and consolidation or pulmonary opacities (often with a bilateral and peripheral lung distribution) (31,32). Bernheim et al. have reported the absence of ancillary CT findings such as pleural effusions, lung cavitation, pulmonary nodules, and lymphadenopathy (31,32). Bai et al. have assessed the performance of U.S. and Chinese radiologists in differentiating COVID-19 from viral pneumonia on chest CT and found high specificity but moderate sensitivity (38). The British Society of Thoracic Imaging has published reporting guidance and a proforma document (which might help to report findings with speed and accuracy) (39), as well as a teaching library. Its content will be accessible without a log-on via the British Society of Thoracic Imaging website (39). COVID-19–suspected pneumonia is ^18^F-FDG–avid and might be detected as an incidental finding in asymptomatic patients undergoing PET/CT (40). The nuclear medicine community should be vigilant about looking for other unexpected scan findings that might reflect the effects of COVID-19 exposure or infection.

## CONCLUSION

COVID-19 has changed the way we work. We should stay informed, support each other, and provide practical solutions for safety and social well-being during these uncertain times. We should adhere to our national and international recommendations. The health-care system and professionals must aim to deliver safe patient care, maintain a safe workplace, and ensure personal wellness.

*Life imposes things on you that you can’t control, but you still have the choice of how you’re going to live through this.*

—Celine Dion

## DISCLOSURE

No potential conflict of interest relevant to this article was reported.
